# Morphological and molecular identification of *Diaporthe* species in south-western China, with description of eight new species

**DOI:** 10.3897/mycokeys.77.59852

**Published:** 2021-01-14

**Authors:** Wenxiu Sun, Shengting Huang, Jiwen Xia, Xiuguo Zhang, Zhuang Li

**Affiliations:** 1 College of Life Sciences, Yangtze University, Jingzhou 434025, Hubei, China Yangtze University Jingzhou China; 2 Shandong Provincial Key Laboratory for Biology of Vegetable Diseases and Insect Pests, College of Plant Protection, Shandong Agricultural University, Taian, Shandong, 271018, China Shandong Agricultural University Taian China

**Keywords:** Diaporthaceae, Diaporthales, phylogeny, taxonomy, 8 new taxa

## Abstract

*Diaporthe* species have often been reported as plant pathogens, endophytes and saprophytes, commonly isolated from a wide range of infected plant hosts. In the present study, twenty strains obtained from leaf spots of twelve host plants in Yunnan Province of China were isolated. Based on a combination of morphology, culture characteristics and multilocus sequence analysis of the rDNA internal transcribed spacer region (ITS), translation elongation factor 1-α (*TEF*), *β*-tubulin (*TUB*), calmodulin (*CAL*), and histone (*HIS*) genes, these strains were identified as eight new species: *Diaporthe camelliae-sinensis*, *D. grandiflori*, *D. heliconiae*, *D. heterostemmatis*, *D. litchii*, *D. lutescens*, *D. melastomatis*, *D. pungensis* and two previously described species, *D. subclavata* and *D. tectonendophytica*. This study showed high species diversity of *Diaporthe* in tropical rain forests and its hosts in south-western China.

## Introduction

*Diaporthe* is a genus in the Diaporthaceae family (Diaporthales), with the asexual morph previously known as *Phomopsis* and type species *Diaporthe eres* Nitschke collected from *Ulmus* sp. in Germany (Nitschke 1870). Nevertheless, with the implementation of “one fungus one name” nomenclature, the generic names *Diaporthe* and *Phomopsis* are no longer used for both morphs of this genus, and Rossman et al. (2015) gave priority to the older name *Diaporthe* Nitschke over *Phomopsis* (Sacc.) Bubák because it was published first, encountered commonly in literatures and represents the majority of species. The sexual morph of *Diaporthe* is characterized by: immersed perithecial ascomata and an erumpent pseudostroma with more or less elongated perithecial necks; unitunicate clavate to cylindrical asci; fusoid, ellipsoid to cylindrical, septate or aseptate, hyaline ascospores, biseriately to uniseriately arranged in the ascus, sometimes having appendages (Udayanga et al. 2011; Senanayake et al. 2017, 2018). The asexual morph is characterized by ostiolate conidiomata, with cylindrical phialides producing three types of hyaline, aseptate conidia (Udayanga et al. 2011; Gomes et al. 2013): type I: α-conidia, hyaline, fusiform, straight, guttulate or eguttulate, aseptate, smooth-walled; type II: β-conidia, hyaline, filiform, straight or hamate, aseptate, smooth-walled, eguttulate; type III: γ-conidia, rarely produced, hyaline, multiguttulate, fusiform to subcylindrical with an acute or rounded apex, while the base is sometimes truncate. The gamma conidia rarely produced and observed, those species described, having a third type of spores are *D. ampelina* (Berk. & M.A. Curtis) R.R. Gomes, Glienke & Crous, *D. cinerascens* Sacc., *D. eres* Nitschke, *D. hongkongensis* R.R. Gomes, C. Glienke & Crous, *D. limonicola* Guarnaccia & Crous, *D. oncostoma* (Duby) Fuckel, *D. perseae* (Zerova) R.R. Gomes, C. Glienke & Crous, *D. raonikayaporum* R.R. Gomes, C. Glienke & Crous (Gomes et al. 2013; Guarnaccia and Crous 2017; Guo et al. 2020).

Currently, more than 1100 epithets of *Diaporthe* are listed in Index Fungorum (http://www.indexfungorum.org/; accessed 1 June 2020), but only one-fifth of these taxa have been studied with molecular data (Guo et al. 2020; Yang et al. 2020; Zapata et al. 2020). They are widely distributed and have a broad range of hosts from economically significant agricultural crops to ornamental plants including *Camellia*, *Castanea*, *Citrus*, *Glycine*, *Helianthus*, *Juglans*, *Persea*, *Pyrus*, *Vaccinium* and *Vitis* (van Rensburg et al. 2006; Santos and Phillips 2009; Crous et al. 2011a, b, 2016; Santos et al. 2011; Thompson et al. 2011; Grasso et al. 2012; Huang et al. 2013; Lombard et al. 2014; Gao et al. 2015, 2016, 2017; Udayanga et al. 2012, 2015; Guarnaccia et al. 2016; Dissanayake et al. 2017; Guarnaccia and Crous 2017; Fan et al. 2018; Senanayake et al. 2018; Guo et al. 2020). Many *Diaporthe* species have been reported as destructive plant pathogens, innocuous endophytes and saprobes (Murali et al. 2006; Udayanga et al. 2012; Gomes et al. 2013; Ménard et al. 2014; Guarnaccia et al. 2016; Torres et al. 2016; Senanayake et al. 2018). However, the biology and lifestyle of some of them remain unclear (Vilka and Volkova 2015).

From previous studies, the methods of species identification and classification in genus *Diaporthe* were based on criteria such as morphological characters like the size and shape of ascomata (Udayanga et al. 2011) and conidiomata (Rehner and Uecker 1994). However, in recent studies, determining species boundaries only by morphological characters was demonstrated to be not always informative due to their variability under changing environmental conditions (Gomes et al. 2013). As for phylogenetic analysis for *Diaporthe* species, the use of a five-locus dataset (ITS-*TUB*-*TEF*-*CAL*-*HIS*) is the optimal combination for species delimitation as revealed by Santos et al. (2017). Thus, in recent years, many *Diaporthe* species have been described based on a polyphasic approach combined with morphological characterization and their host associations (Guarnaccia and Crous 2017; Gao et al. 2017; Yang et al. 2018, 2020; Crous et al. 2020; Dayarathne et al. 2020; Guo et al. 2020; Hyde et al. 2020; Li et al. 2020; Zapata et al. 2020).

In this study, we propose eight novel species and two previously described species of *Diaporthe*, collected in Yunnan Province of China on twelve plant host genera, based on their morphological characters in culture, and molecular phylogenetic analysis.

## Materials and methods

### Isolation and morphological studies

The leaves of samples were collected from Yunnan Province, China. Isolations from surface sterilized leaf tissues were conducted following the protocol of Gao et al. (2014). Tissue fragments (5 × 5 mm) were taken from the margin of leaf lesions and surface-sterilized by consecutively immersing in 75% ethanol solution for 1 min, 5% sodium hypochlorite solution for 30 s, and finally rinsed in sterile distilled water for 1 min. The pieces were dried with sterilized paper towels and transferred on potato dextrose agar (PDA) in petri plates (Cai et al. 2009). All the PDA plates were incubated at biochemical incubator at 25 °C for 2–4 days, and hyphae were picked out of the periphery of the colonies and inoculated onto new PDA plates.

Following 2–3 weeks of incubation, photographs of the fungal colonies were taken at 7 days and 15 days using a Powershot G7X mark II digital camera. Micromorphological characters were observed and documented in distilled water from microscope slides under Olympus SZX10 stereomicroscope and Olympus BX53 microscope, both supplied with Olympus DP80 HD color digital cameras to photo-document fungal structures. All fungal strains were stored in 10% sterilized glycerin at 4 °C for further studies. Voucher specimens were deposited in the Herbarium of Plant Pathology, Shandong Agricultural University (**HSAUP**). Living strain cultures were deposited in the Shandong Agricultural University Culture Collection (**SAUCC**). Taxonomic information on the new taxa was submitted to MycoBank (http://www.mycobank.org).

### DNA extraction and amplification

Genomic DNA was extracted from fungal mycelia on PDA, using a modified cetyltrimethylammonium bromide (CTAB) protocol as described in Guo et al. (2000). The internal transcribed spacer regions with intervening 5.8S nrRNA gene (ITS), part of the beta-tubulin gene region (*TUB*), partial translation elongation factor 1-alpha (*TEF*), histone H3 (*HIS*) and calmodulin (*CAL*) genes were amplified and sequenced by using primers pairs ITS4/ITS5 (White et al. 1990), Bt2a/Bt2b (Glass and Donaldson 1995), EF1-728F/EF1-986R (Carbone and Kohn 1999), CAL-228F/CAL-737R (Carbone and Kohn 1999) and CYLH3F/H3-1b (Glass and Donaldson 1995; Crous et al. 2004), respectively.

PCR was performed using an Eppendorf Master Thermocycler (Hamburg, Germany). Amplification reactions were performed in a 25 μL reaction volume which contained 12.5 μL Green Taq Mix (Vazyme, Nanjing, China), 1 μL of each forward and reverse primer (10 μM) (Biosune, Shanghai, China), and 1 μL template genomic DNA in amplifier, and were adjusted with distilled deionized water to a total volume of 25 μL.

PCR parameters were as follows: 95 °C for 5 min, followed by 35 cycles of denaturation at 95 °C for 30 s, annealing at a suitable temperature for 30 s, extension at 72 °C for 1 min and a final elongation step at 72 °C for 10 min. Annealing temperature for each gene was 55 °C for ITS, 60 °C for *TUB*, 52 °C for *TEF*, 54 °C for *CAL* and 57 °C for *HIS*. The PCR products were visualized on 1% agarose electrophoresis gel. Sequencing was done bi-directionally, conducted by the Biosune Company Limited (Shanghai, China). Consensus sequences were obtained using MEGA 7.0 (Kumar et al. 2016). All sequences generated in this study were deposited in GenBank (Table 1).

### Phylogenetic analyses

Novel sequences generated from twenty strains in this study, and all reference available sequences of *Diaporthe* species downloaded from GenBank were used for phylogenetic analyses. Alignments of the individual locus were determined using MAFFT v. 7.110 by default settings (Katoh et al. 2017) and manually corrected where necessary. To establish the identity of the isolates at species level, phylogenetic analyses were conducted first individually for each locus and then as combined analyses of five loci (ITS, *TUB*, *TEF*, *CAL* and *HIS* regions). Phylogenetic analyses were based on maximum likelihood (ML) and Bayesian inference (BI) for the multi-locus analyses. For BI, the best evolutionary model for each partition was determined using MrModeltest v. 2.3 (Nylander 2004) and incorporated into the analyses. ML and BI were run on the CIPRES Science Gateway portal (https://www.phylo.org/) (Miller et al. 2012) using RaxML-HPC2 on XSEDE (8.2.12) (Stamatakis 2014) and MrBayes on XSEDE (3.2.7a) (Huelsenbeck and Ronquist 2001; Ronquist and Huelsenbeck 2003; Ronquist et al. 2012), respectively. For ML analyses the default parameters were used and BI was carried out using the rapid bootstrapping algorithm with the automatic halt option. Bayesian analyses included five parallel runs of 5,000,000 generations, with the stop rule option and a sampling frequency of 500 generations. The burn-in fraction was set to 0.25 and posterior probabilities (PP) were determined from the remaining trees. The resulting trees were plotted using FigTree v. 1.4.2 (http://tree.bio.ed.ac.uk/software/figtree) and edited with Adobe Illustrator CS5.1. New sequences generated in this study were deposited at GenBank (https://www.ncbi.nlm.nih.gov; Table 1), the alignments and trees were deposited in TreeBASE (http://treebase.org/treebase-web/home.html).

## Results

### Phylogenetic analyses

**Figure 1.**
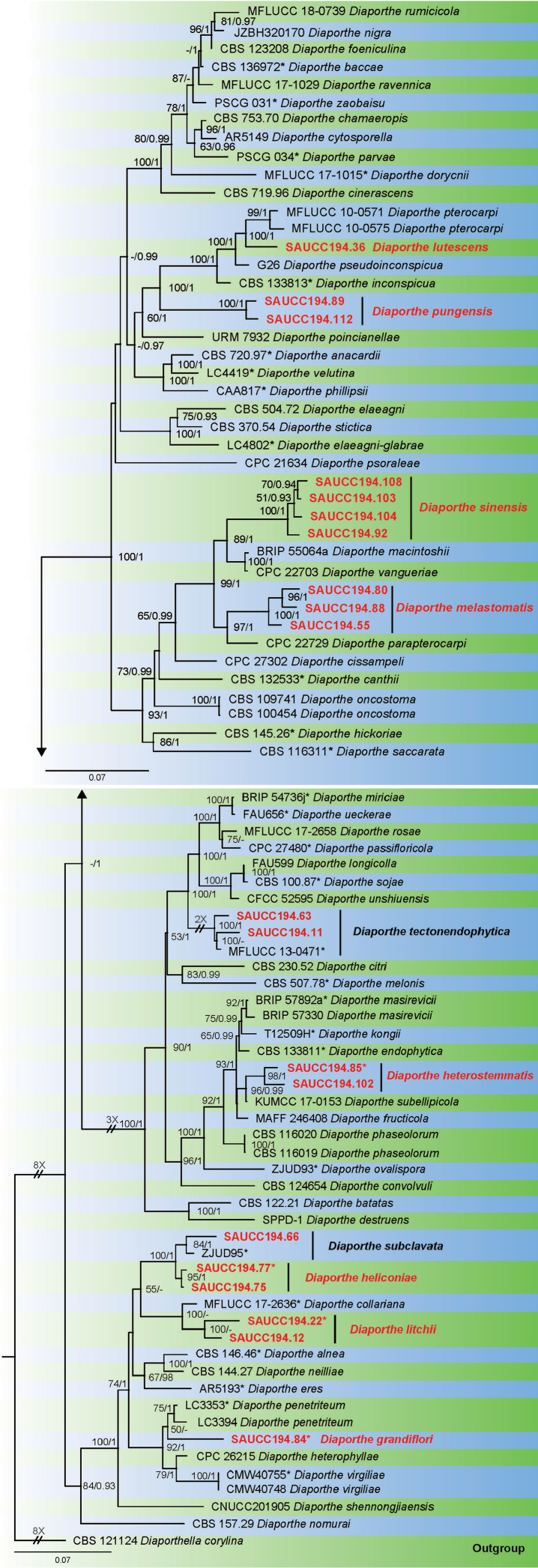
Phylogram of *Diaporthe* based on combined ITS, *TUB*, *TEF*, *CAL* and *HIS* genes. The ML and BI bootstrap support values above 50% and 0.90 BYPP are shown at the first and second position, respectively. Strains marked with “*” are ex-type or ex-epitype. Strains from this study are shown in red. Three branches were shortened to fit the page size – these are indicated by symbol (//) with indication number showing how many times they are shortened.

Twenty fungal strains of *Diaporthe* isolates from 15 plant hosts were sequenced (Table 1). These were analyzed by using multilocus data (ITS, *TUB*, *TEF*, *CAL* and *HIS*) composed of 87 isolates of *Diaporthe*, with *Diaporthella corylina* (CBS 121124) as an outgroup taxon. A total of 2856 characters including gaps were obtained in the phylogenetic analysis, viz. ITS: 1–650, *TUB*: 651–1263, *TEF*: 1264–1705, *CAL*: 1706–2279, *HIS*: 2280–2856. Of these characters, 1395 were constant, 475 were variable and parsimony-uninformative, and 986 were parsimony-informative. For the BI and ML analyses, the substitution model GTR+I+G for ITS, HKY+I+G for *TUB*, *TEF* and *CAL*, GTR+G for *HIS* were selected and incorporated into the analyses. The ML tree topology confirmed the tree topologies obtained from the BI analyses, and therefore, only the ML tree is presented (Fig. 1).

**Table 1.** Species and GenBank accession numbers of DNA sequences used in this study with new sequences in bold.

Species	Strain/Isolate	Host/Substrate	GenBank accession number				
			ITS	*TUB*	*TEF*	*CAL*	*HIS*
*Diaporthe alnea*	CBS 146.46*	*Alnus* sp.	KC343008	KC343976	KC343734	KC343250	KC343492
*D. anacardii*	CBS 720.97*	*Anacardium occidentale*	KC343024	KC343992	KC343750	KC343266	KC343508
*D. baccae*	CBS 136972*	*Vaccinium corymbosum*	KJ160565	–	KJ160597	–	–
*D. batatas*	CBS 122.21	*Ipomoea batatas*	KC343040	KC344008	KC343766	KC343282	KC343524
***D. camelliae-sinensis***	SAUCC194.92*	***Camellia sinensis***	**MT822620**	**MT855817**	**MT855932**	**MT855699**	**MT855588**
	**SAUCC194.103**	***Castanea mollissima***	**MT822631**	**MT855828**	**MT855943**	**MT855710**	**MT855599**
	**SAUCC194.104**	***Castanea mollissima***	**MT822632**	**MT855829**	**MT855944**	**MT855711**	**MT855600**
	**SAUCC194.108**	***Machilus pingii***	**MT822636**	**MT855833**	**MT855948**	**MT855715**	**MT855603**
*D. canthii*	CBS 132533*	*Canthium inerme*	JX069864	KC843230	KC843120	KC843174	–
*D. chamaeropis*	CBS 753.70	*Spartium junceum*	KC343049	KC344017	KC343775	KC343291	KC343533
*D. cinerascens*	CBS 719.96	*Ficus carica*	KC343050	KC344018	KC343776	KC343292	KC343534
*D. cissampeli*	CPC 27302	*Cissampelos capensis*	KX228273	KX228384	–	–	KX228366
*D. citri*	CBS 230.52	*Citrus sinensis*	KC343052	KC344020	KC343778	KC343294	KC343536
*D. collariana*	MFLUCC 17-2636*	*Magnolia champaca*	MG806115	MG783041	MG783040	MG783042	–
*D. convolvuli*	CBS 124654	*Convolvulus arvensis*	KC343054	KC344022	KC343780	KC343296	KC343538
*D. cytosporella*	AR 5149	*Citrus sinensis*	KC843309	KC843223	KC843118	KC843143	–
*D. destruens*	SPPD-1	*Solanum tuberosum*	JN848791	JX421691	–	–	–
*D. dorycnii*	MFLU 17-1015*	*Dorycnium hirsutum*	KY964215	KY964099	KY964171	–	–
*D. elaeagni*	CBS 504.72	*Elaeagnus* sp.	KC343064	KC344032	KC343790	KC343306	KC343548
*D. elaeagni-glabrae*	LC4802*	*Elaeagnus glabra*	KX986779	KX999212	KX999171	KX999281	KX999251
*D. endophytica*	CBS 133811*	*Schinus terebinthifolius*	KC343065	KC344033	KC343791	KC343307	KC343549
*D. eres*	AR5193*	*Ulmus laevis*	KJ210529	KJ420799	KJ210550	KJ434999	KJ420850
*D. foeniculina*	CBS 123208	*Foeniculum vulgare*	KC343104	KC344072	KC343830	KC343346	KC343588
*D. fructicola*	MAFF 246408	*Passiflora edulis*	LC342734	LC342736	LC342735	LC342738	LC342737
***D. grandiflori***	SAUCC194.84*	***Heterostemma grandiflorum***	**MT822612**	**MT855809**	**MT855924**	**MT855691**	**MT855580**
***D. heliconiae***	**SAUCC194.75**	***Heliconia metallica***	**MT822603**	**MT855800**	**MT855915**	**MT855682**	**MT855571**
	SAUCC194.77*	***Heliconia metallica***	**MT822605**	**MT855802**	**MT855917**	**MT855684**	**MT855573**
*D. heterophyllae*	CPC 26215	*Acacia heterophylla*	MG600222	MG600226	MG600224	MG600218	MG600220
***D. heterostemmatis***	SAUCC194.85*	***Heterostemma grandiflorum***	**MT822613**	**MT855810**	**MT855925**	**MT855692**	**MT855581**
	**SAUCC194.102**	***Camellia sinensis***	**MT822630**	**MT855827**	**MT855942**	**MT855709**	**MT855598**
*D. hickoriae*	CBS 145.26*	*Carya glabra*	KC343118	KC344086	KC343844	KC343360	KC343602
*D. inconspicua*	CBS 133813*	*Maytenus ilicifolia*	KC343123	KC344091	KC343849	KC343365	KC343607
*D. kongii*	T12509H*	*Helianthus annuus*	JF431301	KJ197272	JN645797	–	–
***D. litchii***	**SAUCC194.12**	***Elaeagnus conferta***	**MT822540**	**MT855737**	**MT855854**	**MT855625**	**MT855509**
	SAUCC194.22*	***Litchi chinensis***	**MT822550**	**MT855747**	**MT855863**	**MT855635**	**MT855519**
*D. longicolla*	FAU599	*Glycine max*	KJ590728	KJ610883	KJ590767	KJ612124	KJ659188
***D. lutescens***	SAUCC194.36*	***Chrysalidocarpus lutescens***	**MT822564**	**MT855761**	**MT855877**	**MT855647**	**MT855533**
*D. macintoshii*	BRIP 55064a*	*Rapistrum rugostrum*	KJ197289	KJ197269	KJ197251	–	–
*D. masirevicii*	BRIP 57330	*Chrysanthemoides monilifera* subsp. *rotundata*	KJ197275	KJ197255	KJ197237	–	–
	BRIP 57892a*	*Helianthus annuus*	KJ197276	KJ197257	KJ197239	–	–
***D. melastomatis***	SAUCC194.55*	***Melastoma malabathricum***	**MT822583**	**MT855780**	**MT855896**	**MT855664**	**MT855551**
	**SAUCC194.80**	***Millettia reticulata***	**MT822608**	**MT855805**	**MT855920**	**MT855687**	**MT855576**
	**SAUCC194.88**	***Camellia sinensis***	**MT822616**	**MT855813**	**MT855928**	**MT855695**	**MT855584**
*D. melonis*	CBS 507.78*	*Cucumis melo*	KC343142	KC344110	KC343868	KC343384	KC343626
*D. miriciae*	BRIP 54736j*	*Helianthus annuus*	KJ197282	KJ197262	KJ197244	–	–
*D. neilliae*	CBS 144.27	*Spiraea* sp.	KC343144	KC344112	KC343870	KC343386	KC343628
*D. nigra*	JZBH320170	*Ballota nigra*	MN653009	MN887113	MN892277	–	–
*D. nomurai*	CBS 157.29	*Morus* sp.	KC343154	KC344122	KC343880	KC343396	KC343638
*D. oncostoma*	CBS 100454	*Robinia pseudoacacia*	KC343160	KC344128	KC343886	KC343402	KC343644
	CBS 109741	*Robinia pseudoacacia*	KC343161	KC344129	KC343887	KC343403	KC343645
*D. ovalispora*	ZJUD93*	*Citrus limon*	KJ490628	KJ490449	KJ490507	–	KJ490570
*D. parapterocarpi*	CPC 22729	*Pterocarpus brenanii*	KJ869138	KJ869248	–	–	–
*D. parvae*	PSCG 034*	*Pyrus bretschneideri*	MK626919	MK691248	MK654858	–	MK726210
*D. passifloricola*	CPC 27480*	*Passiflora foetida*	KX228292	KX228387	–	–	KX228367
*D. penetriteum*	LC3353*	*Camellia sinensis*	KP714505	KP714529	KP714517	–	KP714493
	LC3394	*Camellia sinensis*	KP267893	KP293473	KP267967	–	KP293544
*D. phaseolorum*	CBS 116019	*Caperonia palustris*	KC343175	KC344143	KC343901	KC343417	KC343659
	CBS 116020	*Aster exilis*	KC343176	KC344144	KC343902	KC343418	KC343660
*D. phillipsii*	CAA 817*	Dead twig	MK792305	MN000351	MK828076	MK883831	MK871445
*D. poincianellae*	URM 7932	*Poincianella pyramidalis*	MH989509	MH989537	MH989538	MH989540	MH989539
*D. pseudoinconspicua*	G26	*Poincianella pyramidalis*	MH122538	MH122524	MH122533	MH122528	MH122517
*D. psoraleae*	CPC 21634	*Psoralea pinnata*	KF777158	KF777251	KF777245	–	–
*D. pterocarpi*	MFLUCC 10-0571	*Pterocarous indicus*	JQ619899	JX275460	JX275416	JX197451	–
	MFLUCC 10-0575	*Pterocarous indicus*	JQ619901	JX275462	JX275418	JX197453	–
***D. pungensis***	**SAUCC194.89**	***Camellia sinensis***	**MT822617**	**MT855814**	**MT855929**	**MT855696**	**MT855585**
	SAUCC194.112*	***Elaeagnus pungens***	**MT822640**	**MT855837**	**MT855952**	**MT855719**	**MT855607**
*D. ravennica*	MFLUCC 17-1029	*Tamarix* sp.	KY964191	KY964075	KY964147	–	–
*D. rosae*	MFLUCC 17-2658	*Rosa* sp.	MG828894	MG843878	–	MG829273	–
*D. rumicicola*	MFLUCC18-0739	*Rumex* sp.	MH846233	MK049555	MK049554	–	–
*D. saccarata*	CBS 116311*	*Protea repens*	KC343190	KC344158	KC343916	KC343432	KC343674
*D. shennongjiaensis*	CNUCC 201905	*Juglans regia*	MN216229	MN227012	MN224672	MN224551	MN224560
*D. sojae*	CBS 100.87*	*Glycine soja*	KC343196	KC344164	KC343922	KC343438	KC343680
*D. stictica*	CBS 370.54	*Buxus sampervirens*	KC343212	KC344180	KC343938	KC343454	KC343696
*D. subclavata*	ZJUD95*	*Citrus unshiu*	KJ490630	KJ490451	KJ490509	–	KJ490572
	**SAUCC194.66**	***Pometia pinnata***	**MT822594**	**MT855791**	**MT855906**	**MT855674**	**MT855562**
*D. subellipicola*	KUMCC 17-0153	on dead wood	MG746632	MG746634	MG746633	–	–
*D. tectonendophytica*	MFLUCC 13-0471*	*Tectona grandis*	KU712439	KU743986	KU749367	KU749354	–
	**SAUCC194.11**	***Elaeagnus conferta***	**MT822539**	**MT855736**	**MT855853**	**MT855624**	**MT855508**
	**SAUCC194.63**	***Pometia pinnata***	**MT822591**	**MT855788**	**MT855903**	**MT855672**	**MT855559**
*D. ueckerae*	FAU656*	*Cucumis melo*	KJ590726	KJ610881	KJ590747	KJ612122	KJ659215
*D. unshiuensis*	CFCC 52595	*Carya illinoensis*	MH121530	MH121607	MH121572	MH121448	MH121488
*D. vangueriae*	CPC 22703	*Vangueria infausta*	KJ869137	KJ869247	–	–	–
*D. velutina*	LC4419*	*Neolitsea* sp.	KX986789	KX999222	KX999181	KX999286	KX999260
*D. virgiliae*	CMW40755*	*Virgilia oroboides*	KP247573	KP247582	–	–	–
	CMW40748	*Virgilia oroboides*	KP247566	KP247575	–	–	–
*D. zaobaisu*	PSCG 031*	*Pyrus bretschneideri*	MK626922	MK691245	MK654855	–	MK726207
*Diaporthella corylina*	CBS 121124	*Corylus* sp.	KC343004	KC343972	KC343730	KC343246	KC343488

Isolates marked with “*” are ex-type or ex-epitype strains.

ML bootstrap support values (≥ 50%) and Bayesian posterior probability (≥ 0.90) are shown as first and second position above nodes, respectively. Based on the five-locus phylogeny and morphology, 20 strains isolated in this study were assigned to 10 species, 8 of them are proposed and described here as new species (Fig. 1). Strains (SAUCC194.92, SAUCC194.103, SAUCC194.104 and SAUCC194.108) are *D. camelliae-sinensis*, strain (SAUCC194.84) – *Diaporthe grandiflori*, strains (SAUCC194.75 and SAUCC194.77) – *D. heliconiae*, strains (SAUCC194.85 and SAUCC194.102) – *D. heterostemmatis*, strains (SAUCC194.12 and SAUCC194.22) – *D. litchii*, strain (SAUCC194.36) – *D. lutescens*, strains (SAUCC194.55, SAUCC194.80 and SAUCC194.88) – *D. melastomatis*, strains (SAUCC194.89 and SAUCC194.112) – *D. pungensis*. One strain (SAUCC194.66) is of a previously described *D. subclavata*, and strains (SAUCC194.11 and SAUCC194.63) – of previously described *D. tectonendophytica*.

### Taxonomy

#### Diaporthe camelliae-sinensis

Taxon classificationFungi

S.T. Huang, J.W. Xia, X.G. Zhang & Z. Li sp. nov.

0DE548B0-B15D-5939-BDFE-852AC226BCB8

837600

Figure 2

##### Etymology.

Named after the host *Camellia sinensis* on which it was collected.

**Figure 2.**
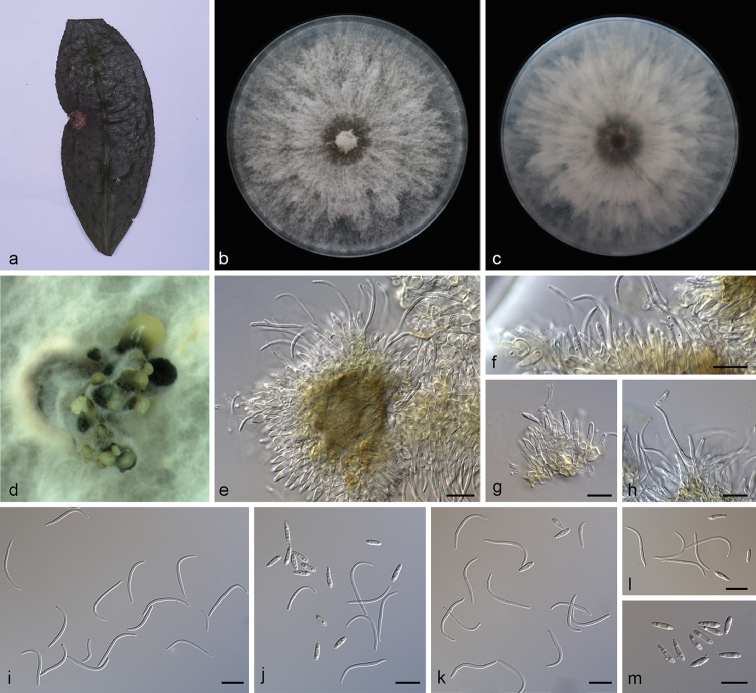
*Diaporthe camelliae-sinensis* (SAUCC194.92) **a** leaf of host plant **b, c** surface (**b**) and reverse (**c**) sides of colony after incubation for 15 days on PDA**d** conidiomata **e–h** conidiophores and conidiogenous cells **i** beta conidia **j–l** alpha conidia and beta conidia **m** alpha conidia. Scale bars: 10 μm (**e–m**).

##### Diagnosis.

*Diaporthe camelliae-sinensis* can be distinguished from the closely related species *D. macintoshii* R.G. Shivas et al. and *D. vangueriae* Crous based on ITS, *TUB* and *TEF* loci. *Diaporthe camelliae-sinensis* differs from *D. macintoshii* in smaller α-conidia and from *D. vangueriae* in shorter β-conidia.

##### Type.

China, Yunnan Province: Xishuangbanna Tropical Botanical Garden, Chinese Academy of Sciences, on infected leaves of *Camellia sinensis*. 19 April 2019, S.T. Huang, HSAUP194.92, holotype, ex-holotype living culture SAUCC194.92.

##### Description.

Asexual morph: Conidiomata pycnidial, multi-pycnidia grouped together, globose, black, erumpent, coated with white hyphae, thick-walled, exuding creamy to yellowish conidial droplets from central ostioles. Conidiophores hyaline, smooth, septate, branched, densely aggregated, cylindrical, straight to sinuous, swelling at the base, tapering towards the apex, 10–15 × 1.5–2 μm. Conidiogenous cells 8.5–12 × 2–2.8 μm, phialidic, cylindrical, terminal, slightly tapering towards the apex. Alpha conidia, hyaline, smooth, aseptate, ellipsoidal to fusoid, 2–4 guttulate, apex subobtuse, base subtruncate, 7.5–10 × 1.8–2.5 µm (mean = 8.5 × 2.2 μm, n = 20). Beta conidia hyaline, aseptate, filiform, sigmoid to lunate, mostly curved through 90–180°, tapering towards the apex, base truncate, 20–30 × 1.2–1.6 µm (mean = 25.6 × 1.3 μm, n = 20). Gamma conidia and sexual morph not observed.

##### Culture characteristics.

Pure culture was isolated by subbing hyphal tips growing from surface sterilized diseased material. Colonies on PDA cover the Petri dish diameter after incubation for 15 days in dark conditions at 25 °C, cottony and radially with abundant aerial mycelium, sparse in the margin. With a tanned concentric ring of dense hyphae, white on surface side, white to pale yellow on reverse side.

##### Additional specimens examined.

China, Yunnan Province: Xishuangbanna Tropical Botanical Garden, Chinese Academy of Sciences, 19 April 2019, S.T. Huang. On infected leaves of *Castanea mollissima*, HSAUP194.103 and HSAUP194.104 paratype, living culture SAUCC194.103 and SAUCC194.104; on diseased leaves of *Machilus pingii*, HSAUP194.108 paratype, living culture SAUCC194.108.

##### Notes.

Four isolates are clustered in a clade distinct from its closest phylogenetic neighbor, *D. macintoshii* and *D. vangueriae*. *Diaporthe camelliae-sinensis* can be distinguished from *D. macintoshii* in ITS, *TUB* and *TEF* loci (23/558 in ITS, 2/463 in *TUB* and 20/328 in *TEF*); from *D. vangueriae* in ITS and *TUB* loci (23/558 in ITS and 1/423 in *TUB*). Morphologically, *Diaporthe camelliae-sinensis* differs from *D. macintoshii* in having guttulate alpha conidia and smaller alpha conidia (7.5–10 × 1.8–2.5 vs. 8.0–11.0 × 2.0–3.0 μm) (Thompson et al. 2015). Furthermore, *Diaporthe camelliae-sinensis* differs from *D. vangueriae* in shorter beta conidia (20–30 × 1.2–1.6 vs. 28–35 × 1.5–2.0 μm) and *D. camelliae-sinensis* can produce alpha conidia, but *D. vangueriae* could not (Crous et al. 2014).

#### Diaporthe grandiflori

Taxon classificationFungi

S.T. Huang, J.W. Xia, X.G. Zhang & Z. Li sp. nov.

1A2EBCE2-3B37-5E62-8686-51F5666ADB69

837591

Figure 3

##### Etymology.

Named after the host *Heterostemma grandiflorum* on which it was collected.

##### Diagnosis.

*Diaporthe grandiflori* can be distinguished from the phylogenetically closely related species *D. penetriteum* Y.H. Gao & L. Cai in larger α-conidia and β-conidia.

**Figure 3.**
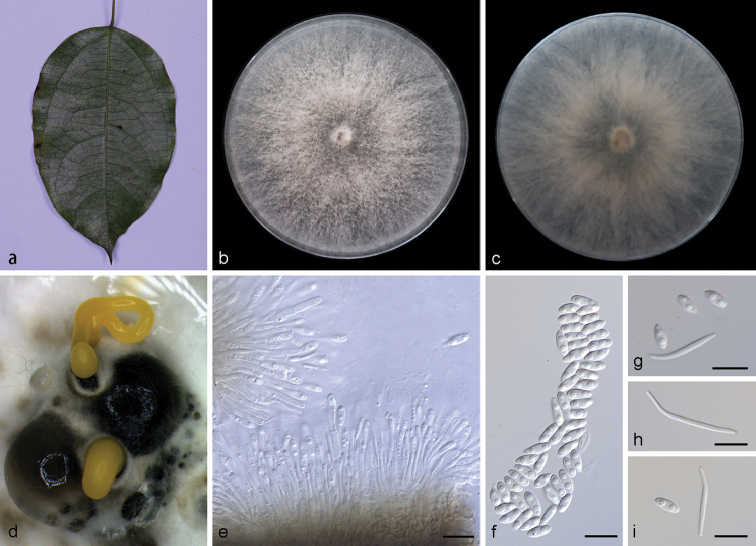
*Diaporthe grandiflori* (SAUCC194.84) **a** leaf of *Heterostemma grandiflorum***b, c** surface (**b**) and reverse (**c**) sides of colony after incubation for 15 days on PDA**d** conidiomata **e** conidiophores and conidiogenous cells **f** alpha conidia **g, i** alpha conidia and beta conidia **h** beta conidia. Scale bars: 10 μm (**e–i**).

##### Type.

China, Yunnan Province: Xishuangbanna Tropical Botanical Garden, Chinese Academy of Sciences, on infected leaves of *Heterostemma grandiflorum*. 19 April 2019, S.T. Huang, HSAUP194.84, holotype, ex-holotype living culture SAUCC194.84.

##### Description.

Asexual morph: Conidiomata pycnidial, subglobose to globose, solitary or aggregated in groups, black, erumpent, coated with white hyphae, thick-walled, exuding golden yellow spiral conidial cirrus from ostiole. Conidiophores hyaline, smooth, septate, branched, densely aggregated, cylindrical, straight to slightly sinuous, 9.5–16.5 × 1.9–2.8 μm. Conidiogenous cells 19.0–22.8 × 1.4–2.4 μm, cylindrical, multi-guttulate, terminal, tapering towards the apex. Alpha conidia abundant in culture, biguttulate, hyaline, smooth, aseptate, ellipsoidal, apex subobtuse, base subtruncate, 6.3–8.3 × 2.8–3.3 µm (mean = 7.5 × 2.9 μm, n = 20). Beta conidia, not numerous, hyaline, aseptate, filiform, slightly curved, tapering towards the apex, 21.5–30.5 × 1.5–2.1 µm (mean = 24.0 × 1.7 μm, n = 20). Gamma conidia not observed. Sexual morph not observed.

##### Culture characteristics.

Pure culture was isolated by subbing hyphal tips growing from surface sterilized plant material. Colonies on PDA cover the Petri dish after 15 days kept in dark conditions at 25 °C, cottony with abundant aerial mycelium, white on surface side, white to grayish on reverse.

##### Notes.

Phylogenetic analysis of a combined five loci showed that *D. grandiflori* (strain SAUCC194.84) formed an independent clade (Fig. 1) and is phylogenetically distinct from *D. penetriteum*. This species can be easily distinguished from *D. penetriteum* by 87 nucleotides difference concatenated alignment (24 in the ITS region, 1 *TUB*, 41 *CAL* and 21 *HIS*). Morphologically, *D. grandiflori* differs from *D. penetriteum* in larger α-conidia (6.3–8.3 × 2.8–3.3 vs. 4.5–5.5 × 1.5–2.5 μm) and longer β-conidia (21.5–30.5 × 1.5–2.1 vs. 16.5–27.5 × 1.0–2.0 μm) (Gao et al. 2016).

#### Diaporthe heliconiae

Taxon classificationFungi

S.T. Huang, J.W. Xia, X.G. Zhang & Z. Li sp. nov.

222ED481-5804-5F9B-A431-9E9326612CFE

837592

Figure 4

##### Etymology.

Named after the host *Heliconia metallica* on which it was collected.

##### Diagnosis.

*Diaporthe heliconiae* can be distinguished from the phylogenetically closely related species *D. subclavata* F. Huang, K.D. Hyde & Hong Y. Li in smaller α-conidia.

**Figure 4.**
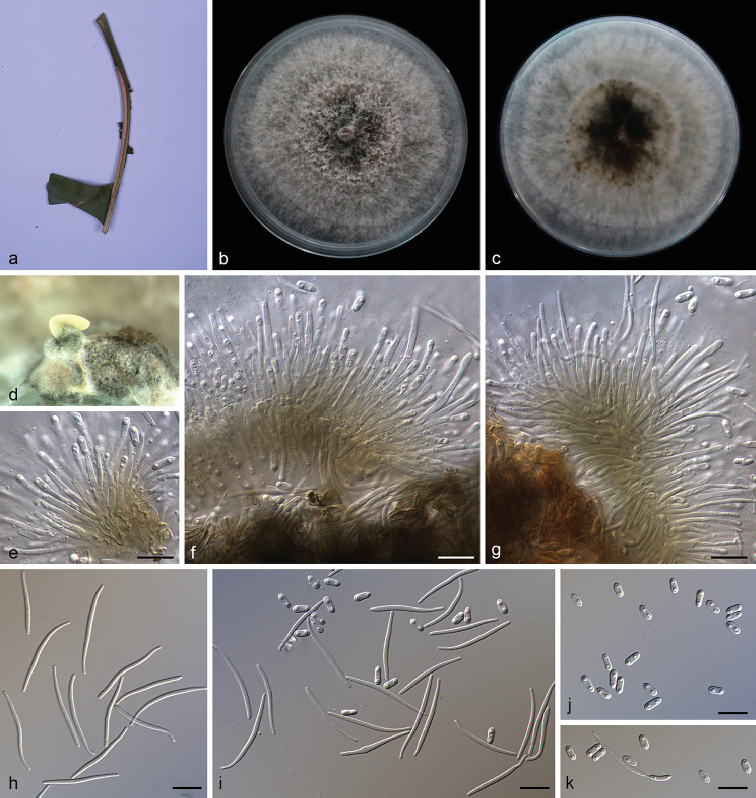
*Diaporthe heliconiae* (SAUCC194.77) **a** petiole of *Heliconia metallica***b, c** surface (**b**) and reverse (**c**) sides of colony after incubation for 15 days on PDA**d** conidiomata on PDA**e–g** conidiophores and conidiogenous cells **h** beta conidia **i** alpha conidia and beta conidia **j** alpha conidia **k** alpha conidia and germinating conidia. All in water. Scale bars: 10 μm (**e–k**).

##### Type.

China, Yunnan Province: Xishuangbanna Tropical Botanical Garden, Chinese Academy of Sciences, on the symptomatic petiole of *Heliconia metallica*. 19 April 2019, S.T. Huang, HSAUP194.77, holotype, ex-holotype living culture SAUCC194.77.

##### Description.

Asexual morph: Conidiomata pycnidial, solitary or aggregated in groups, erumpent, thin-walled, superficial to embedded on PDA, dark brown to black, globose or subglobose, exuding creamy yellowish spiral conidial cirrus from the ostioles. Conidiophores hyaline, aseptate, cylindrical, straight to sinuous, branched, 16.5–25.0 × 1.3–1.8 µm. Alpha conidiogenous cells, cylindric-clavate, terminal, few guttulate, 11.5–18.0 × 1.0–1.5 µm. Beta conidiogenous cells, prismatic, terminal, few guttulate, 10.0–14.1 × 1.0–1.2 µm. Alpha conidia, hyaline, smooth, aseptate, ellipsoidal, 2–4 guttulate, apex subobtuse, base subtruncate, 5.0–6.5 × 2.0–2.5 µm (mean = 6.1 × 2.3 μm, n = 20). Beta conidia hyaline, aseptate, filiform, slightly curved, tapering towards the apex, 25.0–33.5 × 1.0–1.5 µm (mean = 29.4 × 1.3 μm, n = 20). Gamma conidia and sexual morph not observed.

##### Culture characteristics.

Pure culture was isolated by subbing hyphal tips growing from surface sterilized infected plant material. Colonies on PDA cover the Petri dish diameter after incubation for 15 days in dark conditions at 25 °C. Aerial mycelium abundant, cottony, white, dense in the center, sparse near the margin. White on surface side, white to tanned on reverse side.

##### Additional specimen examined.

China, Yunnan Province: Xishuangbanna Tropical Botanical Garden, Chinese Academy of Sciences, on the symptomatic petiole of *Heliconia metallica*. 19 April 2019, S.T. Huang, HSAUP194.75 paratype; living culture SAUCC194.75.

##### Notes.

*Diaporthe heliconiae* clade comprises strains SAUCC194.75 and SAUCC194.77, closely related to *D. subclavata* in the combined phylogenetic tree (Fig. 1). *Diaporthe heliconiae* can be distinguished based on ITS, *TUB* and *HIS* loci from *D. subclavata* (16/489 in ITS, 8/357 in *TUB* and 3/470 in *HIS*). Morphologically, *Diaporthe heliconiae* differs from *D. subclavata* in its smaller α-conidia (5.0–6.5 × 2.0–2.5 vs. 5.5–7.2 × 2.2–2.9 μm). Furthermore, in *Diaporthe heliconiae* β-conidia were obtained size 25.0–33.5 × 1.0–1.5 µm, while in *D. subclavata* β-conidia were not obtained (Huang et al. 2015).

#### Diaporthe heterostemmatis

Taxon classificationFungi

S.T. Huang, J.W. Xia, X.G. Zhang & Z. Li sp. nov.

DF618B9F-FD4E-5BD1-A075-81F27B2272B0

837593

Figure 5

##### Etymology.

Named after the host *Heterostemma grandiflorum* on which it was collected.

##### Diagnosis.

*Diaporthe heterostemmatis* differs from its closest phylogenetic species *D. subellipicola* S.K. Huang & K.D. Hyde in ITS, *TUB* and *TEF* loci based on the alignments deposited in Tree-BASE.

**Figure 5.**
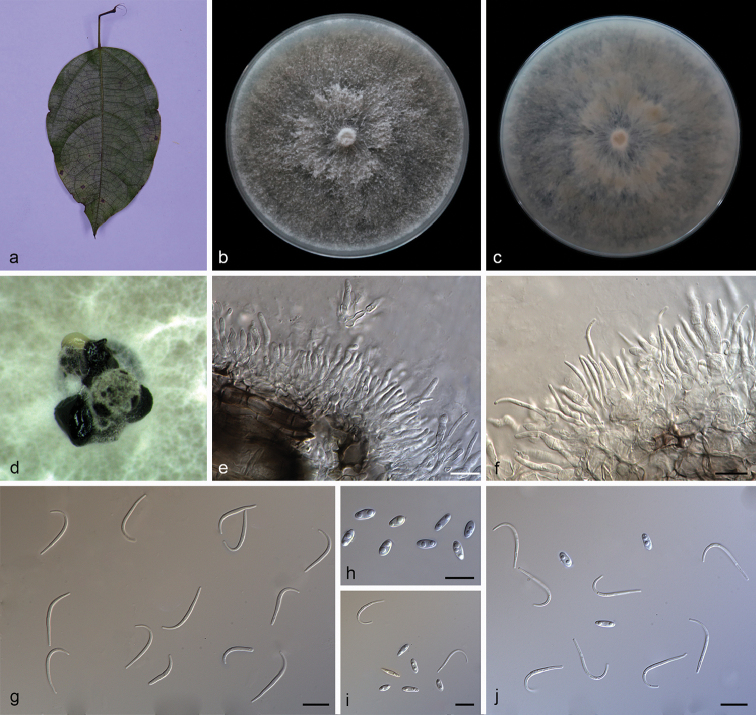
*Diaporthe heterostemmatis* (SAUCC194.85) **a** leaf of host plant **b, c** surface (**b**) and reverse (**c**) sides of colony, after incubation for 15 days on PDA**d** conidiomata on PDA**e, f** conidiophores and conidiogenous cells **g** beta conidia **h** Alpha conidia **i, j** alpha conidia and beta conidia. Scale bars: 10 μm (**e–j**).

##### Type.

China, Yunnan Province: Xishuangbanna Tropical Botanical Garden, Chinese Academy of Sciences, on infected leaves of *Heterostemma grandiflorum*. 19 April 2019, S.T. Huang, HSAUP194.85, holotype, ex-holotype living culture SAUCC194.85.

##### Description.

Asexual morph: Conidiomata pycnidial, 3–5 pycnidia grouped together, globose, black, erumpent, exuding creamy to yellowish conidial droplets from ostioles. Conidiophores hyaline, septate, branched, elliptical or cylindrical, straight to sinuous, 6.5–10.5 × 2.5–4.5 μm. Conidiogenous cells 5.3–11.8 × 1.5–3.2 μm, phialidic, cylindrical, enlarged towards the base, tapering towards the apex, slightly curved, neck up to 5.5 μm long, 2.0 μm wide. Alpha conidia, hyaline, smooth, aseptate, ellipsoidal, biguttulate, apex subobtuse, base subtruncate, 5.8–7.5 × 2.5–3.3 µm (mean = 6.5 × 3.0 μm, n = 20). Beta conidia hyaline, aseptate, filiform, few guttulate, hooked and mostly curved through 90–180°, tapering towards both ends, 16.0–22.7 × 1.0–1.5 µm (mean = 20.4 × 1.2 μm, n = 20). Gamma conidia and sexual morph not observed.

##### Culture characteristics.

Pure culture was isolated by subbing hyphal tips growing from surface sterilized plant material. Colonies on PDA cover the Petri dish diameter after incubation for 15 days in dark conditions at 25 °C. Aerial mycelium white, cottony, feathery, with concentric zonation, white on surface side, pale brown to black on reverse side.

##### Additional specimen examined.

China, Yunnan Province: Xishuangbanna Tropical Botanical Garden, Chinese Academy of Sciences, on infected leaves of *Camellia sinensis*. 19 April 2019, S.T. Huang, HSAUP194.102 paratype; living culture SAUCC194.102.

##### Notes.

This new species is proposed as the molecular data showed it forms a distinct clade with high support (ML/BI=98/1) and it appears most closely related to *D. subellipicola*. *Diaporthe heterostemmatis* can be distinguished from *D. subellipicola* by 57 nucleotides in concatenated alignment, in which 8 were distinct in the ITS region, 28 in the *TUB* region and 21 in the *TEF* region. Morphologically, *D. subellipicola* was observed only on the basis of the sexual morph and culture characteristics (Hyde et al. 2018).

#### Diaporthe litchii

Taxon classificationFungi

S.T. Huang, J.W. Xia, X.G. Zhang, Z. Li sp. nov.

41E024A0-C3FA-5810-A1A6-9A424BDD1756

837595

Figure 6

##### Etymology.

Named after the host *Litchi chinensis* on which it was collected.

##### Diagnosis.

*Diaporthe litchii* differs from *D. collariana* R.H. Perrera & K.D. Hyde in smaller alpha conidia and shorter conidiophores.

##### Type.

China, Yunnan Province: Xishuangbanna Tropical Botanical Garden, Chinese Academy of Sciences, on infected leaves of *Litchi chinensis*. 19 April 2019, S.T. Huang, HSAUP194.22, holotype, ex-holotype living culture SAUCC194.22.

**Figure 6.**
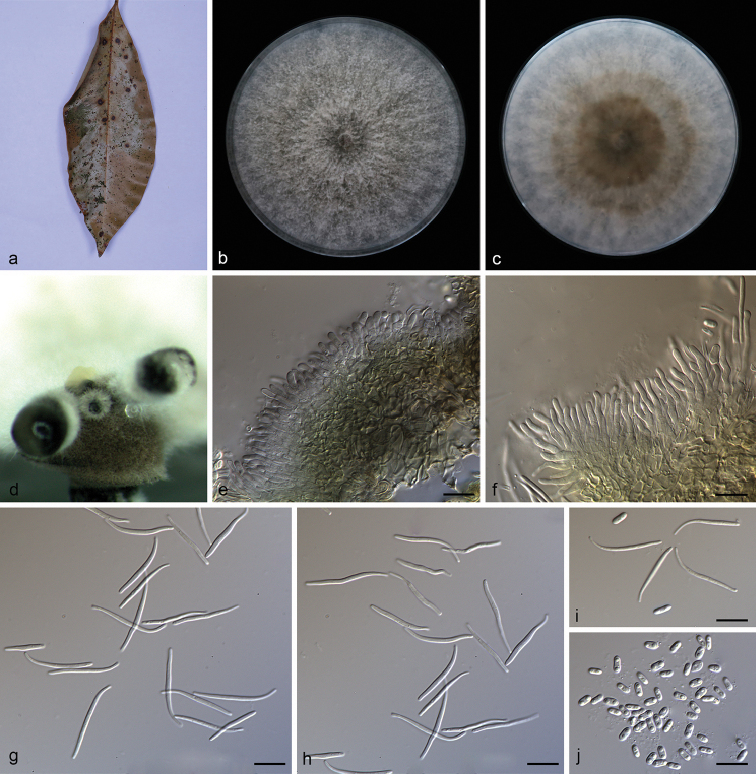
*Diaporthe litchii* (SAUCC194.22) **a** leaf of host plant **b, c** surface (**b**) and reverse (**c**) sides of colony after incubation for 15 days on PDA**d** conidiomata **e, f** conidiophores and conidiogenous cells **g, h** beta conidia **i** alpha conidia and beta conidia **j** alpha conidia. Scale bars: 10 μm (**e–j**).

##### Description.

Asexual morph: Conidiomata pycnidial, 3–5 pycnidia grouped together, globose, black, erumpent, coated with white hyphae, creamy to yellowish conidial droplets exuded from central ostioles. Conidiophores hyaline, branched, densely aggregated, cylindrical, 10.5–15.0 × 1.8–2.5 μm. Conidiogenous cells 7.5–9.5 × 1.5–2.0 μm, cylindrical, terminal, straight to sinuous. Alpha conidia, hyaline, smooth, aseptate, ellipsoidal to fusiform, biguttulate, 3.8–5.0 × 1.5–2.3 µm (mean = 4.7 × 2.0 μm, n = 20). Beta conidia hyaline, aseptate, filiform, few guttulate, slightly curved, tapering towards both ends, 20.0–28.0 × 1.2–1.8 µm (mean = 23.2 × 1.2 μm, n = 20). Gamma conidia and sexual morph not observed.

##### Culture characteristics.

Pure culture was isolated by subbing hyphal tips growing from surface sterilized plant material. Colonies on PDA cover the Petri dish diameter after incubation for 15 days in dark conditions at 25 °C. Aerial mycelium abundant, white, cottony on surface, reverse white to pale brown with two concentric zonation.

##### Additional specimen examined.

China, Yunnan Province: Xishuangbanna Tropical Botanical Garden, Chinese Academy of Sciences, on diseased leaves of *Elaeagnus conferta*. 19 April 2019, S.T. Huang, HSAUP194.12 paratype; living culture SAUCC194.12.

##### Notes.

*Diaporthe litchii* comprises strains SAUCC194.12 and SAUCC194.22 can be distinguished from the closely related species *D. collariana* by 63 nucleotides difference in the concatenated alignment (9 in the ITS region, 34 *TUB*, 5 *TEF* and 15 *CAL*). *Diaporthe litchii* differs from *D. collariana* in smaller alpha conidia (3.8–5.0 × 1.5–2.3 vs. 4.7–5.6 × 1.7–2.2 μm) and shorter conidiophores (10.5–15.0 × 1.8–2.5 vs. 12–20 × 2.4–3.2 μm) (Perera et al. 2018).

#### Diaporthe lutescens

Taxon classificationFungi

S.T. Huang, J.W. Xia, X.G. Zhang & Z. Li sp. nov.

4498E3D7-93FF-51B4-8A51-F81894354C95

837597

Figure 7

##### Etymology.

Named after the host *Chrysalidocarpus lutescens* on which it was collected.

##### Diagnosis.

*Diaporthe lutescens* differs from *D. pterocarpi* (S. Hughes) D. Udayanga et al. and *D. pseudoinconspicua* T.G.L. Oliveira et al. in longer beta conidia and the types of conidia.

**Figure 7.**
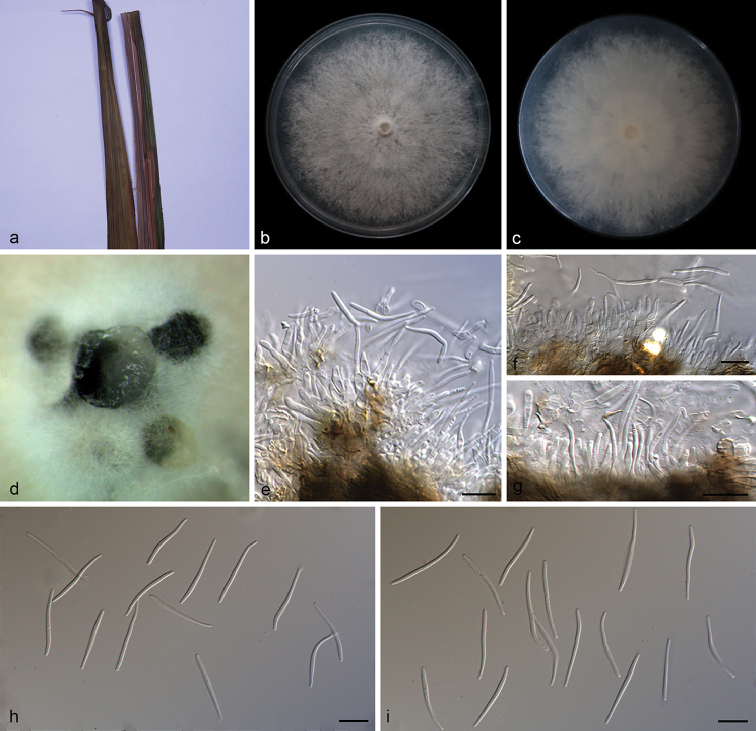
*Diaporthe lutescens* (SAUCC194.36) **a** leaves of host plant **b, c** surface (**b**) and reverse (**c**) sides of colony after incubation for 15 days on PDA**d** conidiomata **e–g** conidiophores and conidiogenous cells **h, i** beta conidia. Scale bars: 10 μm (**e–i**).

##### Type.

China, Yunnan Province: Xishuangbanna Tropical Botanical Garden, Chinese Academy of Sciences, on leaves of *Chrysalidocarpus lutescens*. 19 April 2019, S.T. Huang, HSAUP194.36, holotype, ex-holotype living culture SAUCC194.36.

##### Description.

Asexual morph: Conidiomata pycnidial, scattered or aggregated, black, erumpent, slightly raised above the surface of the culture medium, subglobose, exuding white creamy conidial droplets from central ostioles after 30 days incubation in light condition at 25 °C on PDA; pycnidial wall consists of black to dark brown, thin-walled cells. Conidiophores 10.2–17.0 × 1.8–3.0 μm, hyaline, unbranched, subcylindrical, septate, smooth, straight or slightly curved, obtuse at the apex, widened at base. Conidiogenous cells 5.7–9.1 × 1.4–2.6 μm, phialidic, cylindrical, terminal, straight to sinuous, tapering towards the apex. Beta conidia 20.8–28.8 × 1.2–2.0 μm (mean = 25.3 × 1.4 μm, n = 20), filiform, hyaline, straight or slightly curved, aseptate, base subtruncate, enlarged towards the apex. Alpha conidia and gamma conidia not observed.

##### Culture characteristics.

Pure culture was isolated by subbing hyphal tips growing from surface sterilized infected plant material. Colonies on PDA cover the petri plate diameter after incubation for 15 days in dark conditions at 25 °C, initially white, becoming grayish, reverse pale brown, with concentric rings of dense and sparse hyphae, irregular margin, fluffy aerial mycelium. Pycnidia formed in 15 days.

##### Notes.

From the phylotree, seen on Fig. 1, *Diaporthe lutescens* forms an independent clade and is phylogenetically distinct from *D. pterocarpi* and *D. pseudoinconspicua*. *Diaporthe lutescens* can be distinguished from *D. pterocarpi* in ITS, *TUB*, *TEF* and *CAL* loci by 77 nucleotide differences in concatenated alignment (43 in ITS, 2 in *TUB*, 29 in *TEF* and 17 in *CAL*), and from *D. pseudoinconspicua* in ITS, *TUB*, *TEF*, *CAL* and *HIS* loci by 65 nucleotide differences (18 in ITS, 3 in *TUB*, 23 in *TEF*, 8 in *CAL* and 13 in *HIS*). Moreover, *D. lutescens* differs from *D. pterocarpi* and *D. pseudoinconspicua* in having longer beta conidia (20.8–28.8 × 1.2–2.0 vs. 16.0–23.4 × 1.0–1.4 μm, and 20.8–28.8 × 1.2–2.0 vs. 18.0–21.0 × 1.0–1.5 μm). Furthermore, *Diaporthe pterocarpi* and *D. pseudoinconspicua* can produce α-conidia, but *D. lutescens* cannot (Crous et al. 2018a; Broge et al. 2020).

#### Diaporthe melastomatis

Taxon classificationFungi

S.T. Huang, J.W. Xia, X.G. Zhang & Z. Li sp. nov.

B3D78854-073C-5B4E-BEDA-51AE211D1FC1

837598

Figure 8

##### Etymology.

Named after the host *Melastoma malabathricum* on which it was collected.

##### Diagnosis.

*Diaporthe melastomatis* differs from *D. parapterocarpi* Crous in smaller α-conidia and the types of conidia.

**Figure 8.**
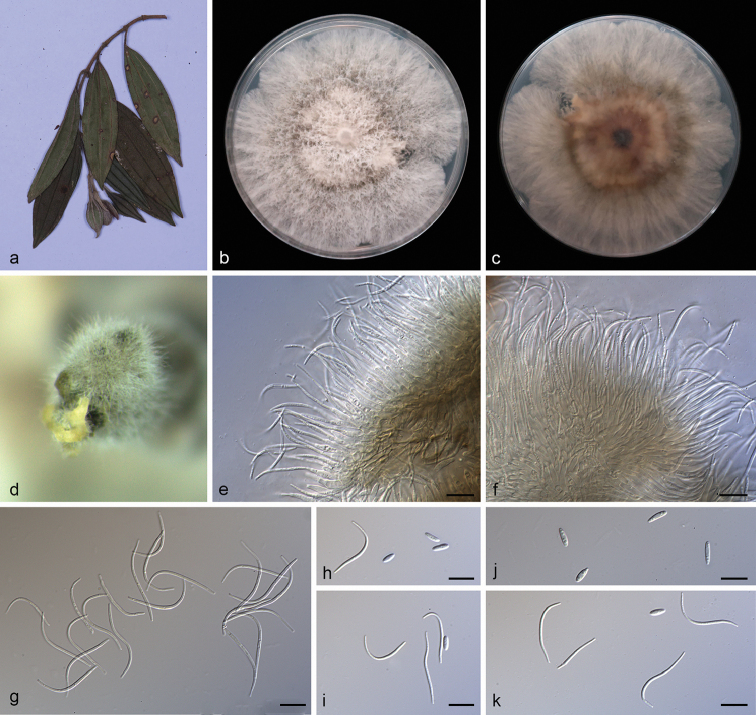
*Diaporthe melastomatis* (SAUCC194.55) **a** branch with leaves of host plant **b, c** surface (**b**) and reverse (**c**) sides of colony after incubation for 15 days on PDA**d** conidiomata **e, f** conidiophores and conidiogenous cells **g** beta conidia **h, i, k** alpha conidia and beta conidia **j** alpha conidia. Scale bars: 10 μm (**e–k**).

##### Type.

China, Yunnan Province: Xishuangbanna Tropical Botanical Garden, Chinese Academy of Sciences, on diseased leaves of *Melastoma malabathricum*. 19 April 2019, S.T. Huang, HSAUP194.55, holotype, ex-holotype living culture, SAUCC194.55.

##### Description.

Asexual morph: Conidiomata pycnidial, subglobose to globose, black, erumpent, coated with white hyphae, thick-walled, yellowish spiral conidial cirrus exuded from ostioles. Conidiophores hyaline, smooth, septate, branched, densely aggregated, cylindric-clavate, straight to slightly sinuous, tapering towards the apex, 14.5–21.0 × 2.0–3.2 μm. Conidiogenous cells 9.5–13.0 × 1.5–2.5 μm, cylindrical, guttulate, terminal, tapering towards the base. Alpha conidia, hyaline, smooth, aseptate, oblong ellipsoidal, 2–4 guttulate, apex subobtuse, base subtruncate, 5.5–8.5 × 1.7–2.5 µm (mean = 6.8 × 2.1 μm, n = 20). Beta conidia abundant in the culture, hyaline, aseptate, filiform, multi-guttulate, sigmoid to lunate, mostly curved through 90–180°, tapering towards both ends, 25.0–33.5 × 1.1–2.0 µm (mean = 27.6 × 1.4 μm, n = 20). Gamma conidia and sexual morph not observed.

##### Culture characteristics.

Pure culture was isolated by subbing hyphal tips growing from surface sterilized diseased material. Colonies on PDA cover the Petri diameter after incubation for 15 days in dark conditions at 25 °C, cottony and lobate with abundant aerial mycelium, hyphae white in the margin on surface side, with pale brown concentric ring of dense hyphae on reverse side.

##### Additional specimens examined.

China, Yunnan Province: Xishuangbanna Tropical Botanical Garden, Chinese Academy of Sciences, 19 April 2019, S.T. Huang. On diseased leaves of *Millettia reticulata*, HSAUP194.80 paratype, living culture SAUCC194.80; on infected leaves of *Camellia sinensis*, HSAUP194.88 paratype, living culture SAUCC194.88.

##### Notes.

*Diaporthe melastomatis* is introduced based on the multi-locus phylogenetic analysis, with three isolates clustering separately in a well-supported clade (ML/BI = 100/1). *Diaporthe melastomatis* is most closely related to *D. parapterocarpi*, but distinguished based on ITS and *TUB* loci from *D. parapterocarpi* by 32 nucleotides difference in the concatenated alignment, in which 20 are distinct in the ITS region, 12 in the *TUB* region. Morphologically, *Diaporthe melastomatis* differs from *D. parapterocarpi* in its smaller alpha conidia (5.5–8.5 × 1.7–2.5 vs. 8.0–10.0 × 2.5–3.0 μm). Furthermore, *Diaporthe melastomatis* can produce beta conidia, but *D. parapterocarpi* cannot (Crous et al. 2014).

#### Diaporthe pungensis

Taxon classificationFungi

S.T. Huang, J.W. Xia, X.G. Zhang, Z. Li sp. nov.

8658054E-C61B-5A39-9B67-DB151DC5ECE2

837599

Figure 9

##### Etymology.

Named after the host *Elaeagnus pungens* on which it was collected.

##### Diagnosis.

*Diaporthe pungensis* differs from its closest phylogenetic species *D. inconspicua* R.R. Gomes et al. and *D. poincianellae* T.G.L. Oloveira et al. in ITS, *TUB*, *TEF*, *CAL* and *HIS* loci based on the alignments deposited in Tree-BASE.

**Figure 9.**
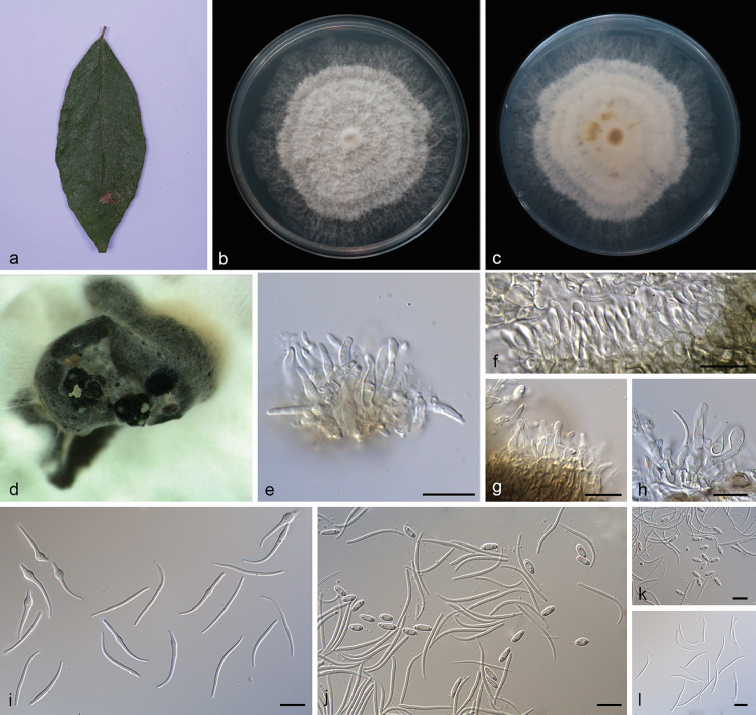
*Diaporthe pungensis* (SAUCC194.112) **a** leaf of host plant **b, c** surface (**b**) and reverse (**c**) sides of colony after incubation for 15 days on PDA**d** conidiomata on PDA**e–h** conidiophores and conidiogenous cells **i, l** beta conidia **j, k** alpha conidia and beta conidia. Scale bars: 10 μm (**e–l**).

##### Type.

China, Yunnan Province: Xishuangbanna Tropical Botanical Garden, Chinese Academy of Sciences, on diseased leaves of *Elaeagnus pungens*. 19 April 2019, S.T. Huang, HSAUP194.112, holotype, ex-holotype living culture SAUCC194.112.

##### Description.

Asexual morph: Conidiomata pycnidial, 3–5 pycnidia grouped together, superficial to embedded on PDA, erumpent, thin-walled, dark brown to black, globose or subglobose, exuding white creamy conidial mass from the ostioles. Conidiophores hyaline, aseptate, cylindrical, smooth, straight to sinuous, unbranched, 11.0–14.5 × 1.5–2.3 µm. Conidiogenous cells phialidic, cylindrical, terminal, 8.0–9.5 × 1.0–2.5 µm. Alpha conidia, hyaline, smooth, aseptate, ellipsoidal to fusoid, 2–3 guttulate, apex subobtuse, base subtruncate, 6.0–8.5 × 2.0–3.3 µm (mean = 6.6 × 2.5 μm, n = 20). Beta conidia hyaline, aseptate, eguttulate, filiform, slightly curved, tapering towards the apex, base truncate, some conidia are in the immature stage swollen in the middle, 24.0–28.9 × 1.0–2.0 µm (mean = 26.9 × 1.4 μm, n = 20). Gamma conidia not observed, sexual morph not observed.

##### Culture characteristics.

Pure culture was isolated by subbing hyphal tips growing from surface sterilized plant material. Colonies on PDA cover the 3/4 of Petri dish diameter after incubation for 15 days in dark conditions at 25 °C, flat, cottony in the center with medium developed aerial mycelium, sparse in the outer region. With several concentric rings of dense and sparse hyphae, irregular margin, white on surface side, white to pale yellow and cinnamon speckle on reverse side.

##### Additional specimen examined.

China, Yunnan Province: Xishuangbanna Tropical Botanical Garden, Chinese Academy of Sciences, on infected leaves of *Camellia sinensis*. 19 April 2019, S.T. Huang, HSAUP194.89 paratype, living culture SAUCC194.89.

##### Notes.

*Diaporthe pungensis* forms a distinct clade with high support (ML/BI = 100/1), and differed with the closely related species (*D. inconspicua* and *D. poincianellae*) on ITS, *TUB*, *CAL* and *HIS* loci (94% in ITS, 92% in *TUB*, 70% in *TEF*, 92% in *CAL* and 92% in *HIS*; and 95% in ITS, 94% in *TUB*, 80% in *TEF*, 94% in *CAL* and 89% in *HIS*, respectively). Moreover, *Diaporthe pungensis* differs from *D. inconspicua*, in having guttulate of alpha conidia, and having larger alpha conidia (6.0–8.5 × 2.0–3.3 vs. 5.5–6.5 × 1.5–2 μm) (Bezerra et al. 2018). Furthermore, *Diaporthe pungensis* can produce two types of conidia (α-conidia and β-conidia), but *D. poincianellae* only produce a α-conidia(Crous et al. 2018b).

#### Diaporthe subclavata

Taxon classificationFungi

F. Huang, K.D. Hyde & H.Y. Li, Fung. Biol. 119: 343, 2015

450781D4-6E99-5B16-882D-25C1993AA7AD

Figure 10

##### Description.

Asexual morph: Conidiomata pycnidial, multi-pycnidia grouped together, globose, black, erumpent, coated with white hyphae, creamy to yellowish conidial droplets exuded from central ostioles. Conidiophores hyaline, densely aggregated, cylindrical, straight to sinuous, tapering towards the apex, 13.5–23.0 × 2.0–3.0 μm. Alpha conidiogenous cells 7.0–10 × 1.8–2.5 μm, cylindrical, terminal, slightly curved. Beta conidiogenous cells 10.5–13.5 × 0.9–1.5 μm, cylindrical, hyaline, tapering towards the apex. Alpha conidia, hyaline, smooth, aseptate, ellipsoidal, multi-guttulate, apex subobtuse, base subtruncate, 4.7–5.8 × 2.4–2.9 µm (mean = 5.3 × 2.6 μm, n = 20). Beta conidia hyaline, aseptate, filiform, few guttulate, slightly curved, tapering towards the both ends, 25.5–32.0 × 1.0–1.6 µm (mean = 27.5 × 1.3 μm, n = 20). Gamma conidia and sexual morph not observed.

**Figure 10.**
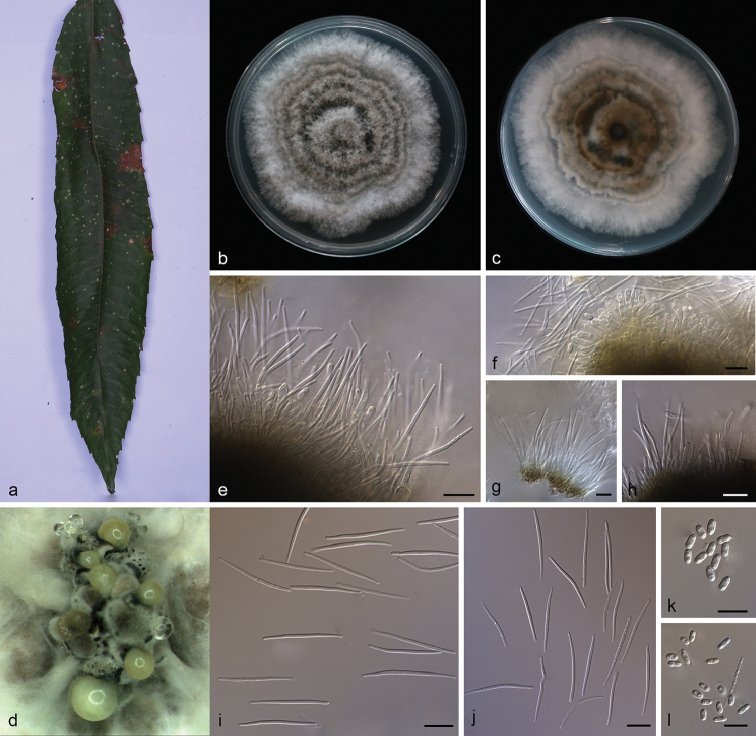
*Diaporthe subclavata* (SAUCC194.66) **a** leaf of *Pometia pinnata***b, c** surface (**b**) and reverse (**c**) sides of colony after incubation for 15 days on PDA**d** conidiomata **e–h** conidiophores and conidiogenous cells **i, j** Beta conidia **k, l** Alpha conidia. Scale bars: 10 μm (**e–l**).

##### Culture characteristics.

Pure culture was isolated by subbing hyphal tips growing from surface sterilized diseased material. Colonies on PDA cover the Petri dish diameter after incubation for 15 days in dark conditions at 25 °C. Aerial mycelium white, cottony, feathery, with concentric zonation, white on surface side, pale brown to black on reverse side.

##### Specimen examined.

China, Yunnan Province: Xishuangbanna Tropical Botanical Garden, Chinese Academy of Sciences, on infected leaves of *Pometia pinnata*. 19 April 2019, S.T. Huang, HSAUP194.66, living culture SAUCC194.66.

##### Notes.

*Diaporthe subclavata* was originally described from the leaf with citrus scab of *Citrus unshiu* in Fujian Province, China (Huang et al. 2015). In the present study, isolated strain SAUCC194.66 from symptomatic leaves of *Pometia pinnata* was congruent with *D. subclavata* based on morphology and DNA sequences data (Fig. 1). We therefore present a description and illustration of *D. subclavata* as a known species for this clade, found on new host.

#### Diaporthe tectonendophytica

Taxon classificationFungi

M. Doilom, A. J. Dissanayake & K.D. Hyde, Fung. Div., 82: 163, 2016

10FD94BE-E177-5477-9071-1000FAC80F69

Figure 11

##### Description.

Asexual morph: Conidiomata pycnidial, aggregated, brownish to black, erumpent, subglobose, exuding white creamy conidial droplets from central ostioles after being kept for 30 days in light at 25 °C. Conidiophores 17.4–35.0 × 2.2–3.5 μm, hyaline, branched, subcylindrical, septate, straight or slightly curved, guttulate. Conidiogenous cells 11.3–15.0 × 1.7–2.5 μm (mean = 12.3 × 2.1 μm, n = 20), cylindric-clavate, hyaline, straight to slightly sinuous, tapering towards the apex. Beta conidia 25.0–31.8 × 0.9–1.8 μm (mean = 28.2 × 1.2 μm, n = 20), filiform, hyaline, guttulate, aseptate, hooked and mostly curved through 90–180°, swollen in the middle. Alpha conidia and Gamma conidia not observed.

**Figure 11.**
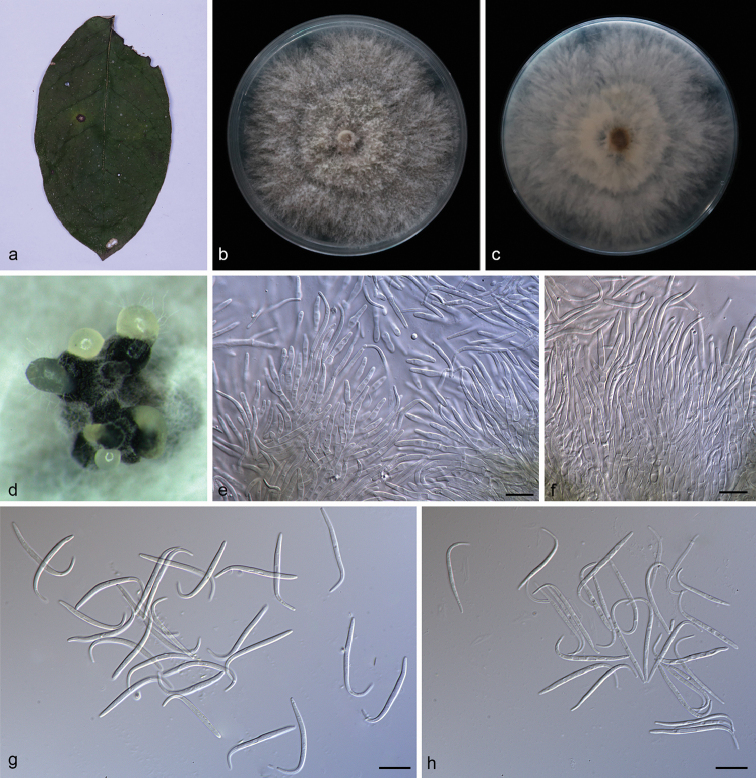
*Diaporthe tectonendophytica* (SAUCC194.11) **a** leaf of host plant **b, c** surface (**b**) and reverse (**c**) side of colony after incubation for 15 days on PDA**d** conidiomata on PDA**e, f** conidiophores and conidiogenous cells **g, h** beta conidia. Scale bars: 10 μm (**e–h**).

##### Culture characteristics.

Pure culture was isolated by subbing hyphal tips growing from surface sterilized diseased material. Colonies on PDA cover the Petri dish diameter after incubation for 15 days in dark conditions at 25 °C, aerial mycelium abundant, white to grayish on surface side, pale yellow on reverse with concentric zonation. Pycnidia are formed on 15^th^ day or later.

##### Specimens examined.

China, Yunnan Province: Xishuangbanna Tropical Botanical Garden, Chinese Academy of Sciences, 19 April 2019, S.T. Huang. On diseased leaves of *Elaeagnus conferta* HSAUP194.11, living culture SAUCC194.11; on diseased leaves of *Pometia pinnata* HSAUP194.63, living culture SAUCC194.63.

##### Notes.

*Diaporthe tectonendophytica* was originally described from the asymptomatic branches of *Tectona grandis* in Thailand (Doilom et al. 2017). In the present study, two strains (SAUCC194.11 and SAUCC194.63) from symptomatic leaves of *Elaeagnus conferta* and *Pometia pinnata* were congruent with *D. tectonendophytica* based on morphology and DNA sequences data (Fig. 1). We therefore describe *D. tectonendophytica* as a known species for this clade.

## Discussion

In the current study, 87 reference sequences (including an outgroup taxon) were selected based on BLAST searches of NCBIs GenBank nucleotide database and were included in the phylogenetic analyses (Table 1). Phylogenetic analyses based on five combined loci (ITS, *TUB*, *TEF*, *CAL* and *HIS*), as well as morphological characters of the non-sexual morph obtained in culture, contributed to knowledge of the diversity of *Diaporthe* species in Yunnan Province. Based on a large set of freshly collected specimens from Yunnan province, China, 20 strains of *Diaporthe* species were isolated from 12 host genera (Table 1). As a result, eight new species are proposed: *Diaporthe camelliae-sinensis*, *D. grandiflori*, *D. heliconiae*, *D. heterostemmatis*, *D. litchii*, *D. lutescens*, *D. melastomatis*, *D. pungensis* and two previously described species were described and illustrated, *D. subclavata* and *D. tectonendophytica*.

Previously, species identification of *Diaporthe* was largely referred to the assumption of host-specificity, leading to the proliferation of names (Gomes et al. 2013). However, based on a polyphasic approach and known morphology, more than one species of *Diaporthe* can colonize a single host, while one species can be associated with different hosts (Gomes et al. 2013; Gao et al. 2017; Guarnaccia and Crous 2017; Guarnaccia et al. 2018; Guo et al. 2020). Our study can well support this phenomenon. On the one hand, *Diaporthe grandiflori* (SAUCC194.84) and *D. heterostemmatis* (SAUCC194.85) were collected from *Heterostemma grandiflorum*; *D. camelliae-sinensis* (SAUCC194.92), *D. heterostemmatis* (SAUCC194.102), *D. melastomatis* (SAUCC194.88), and *D. pungensis* (SAUCC194.89) and were isolated from *Camellia sinensis*; *D. litchii* (SAUCC194.12) and *D. tectonendophytica* (SAUCC194.11) were known on *Elaeagnus conferta*. On the other hand, the species of *D. camelliae-sinensis* collected from three hosts (*Camellia sinensis*, *Castanea mollissima*, *Machilus pingii*) *D. melastomatis* sampled from three hosts (*Camellia sinensis*, *Melastoma malabathricum*, *Millettia reticulata*) and *D. litchii* sampled from two hosts (*Elaeagnus conferta*, *Litchi chinensis*). These studies revealed a high diversity of *Diaporthe* species from different hosts. The descriptions and molecular data of *Diaporthe* represent an important resource for plant pathologists, plant quarantine officials and taxonomists.

## Supplementary Material

XML Treatment for Diaporthe camelliae-sinensis

XML Treatment for Diaporthe grandiflori

XML Treatment for Diaporthe heliconiae

XML Treatment for Diaporthe heterostemmatis

XML Treatment for Diaporthe litchii

XML Treatment for Diaporthe lutescens

XML Treatment for Diaporthe melastomatis

XML Treatment for Diaporthe pungensis

XML Treatment for Diaporthe subclavata

XML Treatment for Diaporthe tectonendophytica
